# Exploring the Degradation of Ibuprofen by *Bacillus thuringiensis* B1(2015b): The New Pathway and Factors Affecting Degradation

**DOI:** 10.3390/molecules22101676

**Published:** 2017-10-09

**Authors:** Ariel Marchlewicz, Urszula Guzik, Wojciech Smułek, Danuta Wojcieszyńska

**Affiliations:** 1 Department of Biochemistry, Faculty of Biology and Environmental Protection, University of Silesia in Katowice, Jagiellońska 28, 40-032 Katowice, Poland; amarchlewicz@us.edu.pl (A.M.); urszula.guzik@us.edu.pl (U.G.); 2 Institute of Chemical Technology and Engineering, Poznan University of Technology, Berdychowo 4, 60-965 Poznan, Poland; wojciech.smulek@gmail.com

**Keywords:** biodegradation, ibuprofen, enzymes, hydroxyibuprofen, *Bacillus*

## Abstract

Ibuprofen is one of the most often detected pollutants in the environment, particularly at landfill sites and in wastewaters. Contamination with pharmaceuticals is often accompanied by the presence of other compounds which may influence their degradation. This work describes the new degradation pathway of ibuprofen by *Bacillus thuringiensis* B1(2015b), focusing on enzymes engaged in this process. It is known that the key intermediate which transformation limits the velocity of the degradation process is hydroxyibuprofen. As the degradation rate also depends on various factors, the influence of selected heavy metals and aromatic compounds on ibuprofen degradation by the B1(2015b) strain was examined. Based on the values of non-observed effect concentration (NOEC) it was found that the toxicity of tested metals increases from Hg(II) < Cu(II) < Cd(II) < Co(II) < Cr(VI). Despite the toxic effect of metals, the biodegradation of ibuprofen was observed. The addition of Co^2+^ ions into the medium significantly extended the time necessary for the complete removal of ibuprofen. It was shown that *Bacillus thuringiensis* B1(2015b) was able to degrade ibuprofen in the presence of phenol, benzoate, and 2-chlorophenol. Moreover, along with the removal of ibuprofen, degradation of phenol and benzoate was observed. Introduction of 4-chlorophenol into the culture completely inhibits degradation of ibuprofen.

## 1. Introduction

The increased use of non-steroidal anti-inflammatory drugs has contributed to their presence in natural waters and wastewaters. Ibuprofen is one of the active compounds more frequently detected in the influents and effluents of wastewater treatment plants. In the final effluents and surface water concentration of ibuprofen varies from 0.018 to 4.24 μg/L and from 0.042 to 1.26 µg/L, respectively [1,2,3]. Although ibuprofen is considered as one of the most easily transformed pharmaceuticals with removal efficiencies above 90% its metabolites are still observed in the effluents of biological wastewater treatment system [1,2,3,4]. A large number studies are related to the physico-chemical mechanisms of ibuprofen degradation while there is very little information about the microbiological decomposition of this drug. Murdoch and Hay [5,6,7] isolated and described two bacterial strains able to utilize ibuprofen and proposed degradation pathways. In *Sphingomonas* sp. strain Ibu-2 ibuprofen degradation occurs through its hydroxylation to isobutylocatechol, which is then cleaved to 5-formyl-2-hydroxy-7-methylocta-2,4-dienoic acid, oxidized to 2-hydroxy-5-isobutylhexa-2,4-dienedioic acid [5,6]. The GC-MS analysis showed that in *Variovorax* Ibu-1 ibuprofen is transformed to trihydroxyibuprofen which probably is the dead-end metabolite [5,6,7]. Almeida et al. [8] described *Patulibacter* sp. Strain I11 able to degrade paracetamol under cometabolic conditions. In the studied strain, proteomic analysis showed the presence of acyl-CoA synthetase and protein containing a Rieske (2Fe-2S) iron-sulfur domain. Such enzymes were also detected in *Sphingomonas* Ibu-2. It may indicate that these proteins have a similar role in ibuprofen degradation [8]. Zwiener et al. [9] and Quintana et al. [10] observed other metabolites during ibuprofen degradation in bioreactors. These authors suggested that the first reaction of ibuprofen utilization is the transformation of the aliphatic chain of ibuprofen. Quintana et al. [10] identified two isomers of hydroxyibuprofen which were further metabolized. Zwiener et al. [9] also detected hydroxyibuprofen as a metabolite under oxic conditions. Moreover, under anoxic conditions, carboxyhydratropic acid appeared. Carboxyibuprofen was identified during ibuprofen degradation under oxic and anoxic conditions [9].

The wastewaters frequently contain a mixture of aromatic compounds, organic solvents, heavy metals or synthetic polymers which may influence the efficiency of biodegradation processes [11,12]. It is suggested that heavy metals, such as Cr(VI) or Hg(II), inhibit the assimilation of aromatic compounds by the interaction with essential cellular components. These metals are known to damage the cell membranes, nucleic acids, and enzymes engaged in the biodegradation processes [12,13,14]. As a consequence, extended acclimation periods and reduced biodegradation rates are observed [11,13,15]. The presence of organic compounds with structural similarity to the target compound may cause the competition for the active centers of the key enzymes [16,17]. However, these compounds may also induce the same metabolic pathways and in this way stimulate degradation of the target compound [17,18]. Moreover, the concomitant organic contaminations could be an additional carbon and energy source increasing the degradation efficiency [16,17].

Our previous study showed that *Bacillus thuringiensis* B1(2015b) degrades ibuprofen both under monosubstrate and cometabolic conditions [19,20]. For that reason the objectives of this study were: (1) to examine the influence of environmental factors of pH and temperature on the ibuprofen degradation rate; (2) to investigate the effect of potential co-pollutants: heavy metals and selected aromatic compounds on ibuprofen degradation; and (3) to identify metabolites formed during ibuprofen decomposition and the enzymes engaged in ibuprofen degradation by Gram-positive *Bacillus thuringiensis* strain B1(2015b).

## 2. Results and Discussion

### 2.1. Factors Affecting Ibuprofen Degradation

Ibuprofen degradation depends on many environmental factors which may influence both: the processes directly connected with ibuprofen decomposition (e.g., enzymes of degradation pathway) and the whole cell metabolism. For example, an increase in the metabolism rate at higher temperature may cause biomass growth. In consequences it should lead to increase of ibuprofen degradation rate. On the other hand, factors limiting the growth of microorganisms through the inhibition of the main metabolic pathway, may indirectly influence ibuprofen decomposition. For that reason the knowledge about influence of environmental factors on ibuprofen degradation may allow predicting the degradation ability of the examined strain in the environment.

#### 2.1.1. Influence of pH and Temperature on Ibuprofen Degradation

**Figure 1:**
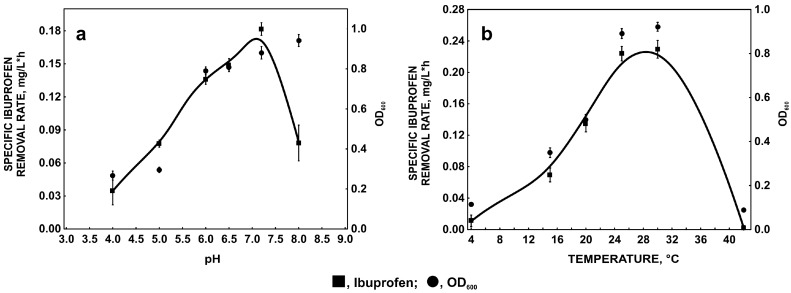
Influence of pH (**a**) and temperature (**b**) on the specific ibuprofen removal rate and bacterial growth expressed as an optical density at 600 nm. The data points represent the average of three independent experiments.

**Figure 2:**
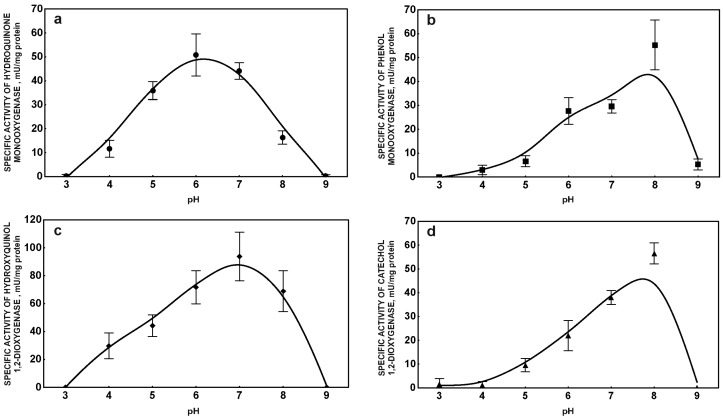
Influence of pH on the activity of hydroquinone monooxygenase (**a**); phenol monooxygenase (**b**); hydroxyquinol 1,2-dioxygenase (**c**); and catechol 1,2-dioxygenase (**d**). The data points represent the average of three independent experiments.

pH is a major factor which significantly influences bacterial cell morphology, membrane characteristic and microbial activity [21]. Additionally, pH may impact biosorption, toxicity, and ionization of pharmaceuticals present in the environment [22,23]. In the present studies *Bacillus thuringiensis* B1(2015b) degraded ibuprofen with the highest removal efficiency at pH 7.2. Simultaneously, significant inhibition of the removal rate at 8.0 pH was observed despite the high amount of bacteria (Figure 1a). In some cases a lower degradation rate may be connected with the ionic form of the molecule at alkaline pH. Under these conditions the surface of the bacterial cell is negatively charged, leading to lower electrostatic interaction between negatively-charged molecules and binding sites of the biomass surface. Ibuprofen has a pKa of 4.3 and at pH higher than 6.5 more than 99% of this drug is present in anionic form. Therefore, at pH 7.2 and 8.0 differences in degradation efficiency may not be connected with different ionic forms of ibuprofen. Another reason is that the observed difference may be a functional state of protein engaged in the degradation process. At low pH (4.0–5.0) ibuprofen is present in an uncharged form and interacts with the surface of bacteria cells [23]. This may suggest easier and faster degradation of ibuprofen, however, in the uncharged form this compound is more toxic to organisms [22]. Our results seem to confirm this evidence. We observed a lower ibuprofen degradation rate and low optical density of bacteria culture at acidic pH (Figure 1a). A low ibuprofen degradation rate at 8.0 pH was probably connected with the activity of degradative enzymes. Although maximal activity of phenol monooxygenase and catechol 1,2-dioxygenase at this pH was observed, hydroxyquinol 1,2-dioxygenase showed 74% of its maximal activity. Hydroquinone monooxygenase had maximal activity at pH 6.0 while, at pH 8.0, significant decrease of the enzyme activity was observed (Figure 2a–d). Hydroquinone monooxygenase is a FAD-dependent monooxygenase. Above pH 7.5 this enzyme easily loses the FAD molecule that may significantly decrease enzyme activity at pH 8.0 [24]. This may suggest that the activity of this enzyme limits the ibuprofen removal rate.

**Figure 3:**
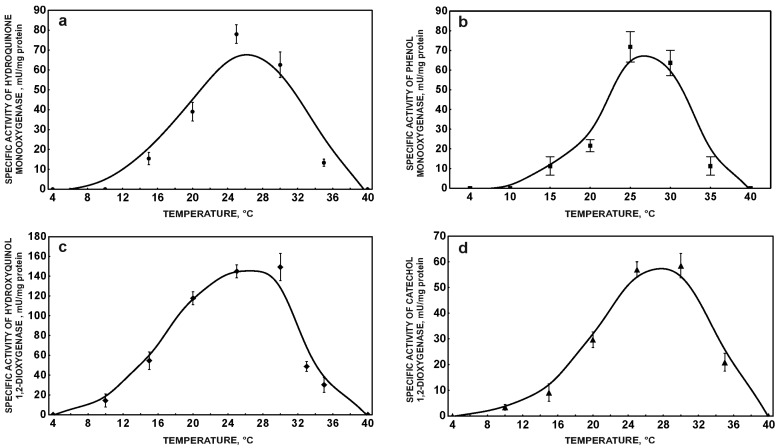
Influence of temperature on the activity of hydroquinone monooxygenase (**a**); phenol monooxygenase (**b**); hydroxyquinol 1,2-dioxygenase (**c**); and catechol 1,2-dioxygenase (**d**). The data points represent the average of three independent experiments.

It is well known that one of the most important factors that influences biodegradation processes is temperature. According to van’t Hoff’s principle the speed of a chemical reaction increases by a factor of 2–4 for a 10 °C rise in temperature [25]. However, in biological systems, the impact of temperature on biodegradation process is more complicitous. It is connected with the relationship between the functioning of the cell membrane and temperature. Too high a temperature may cause denaturation of membrane-bound proteins, whereas at too low a temperature the membrane phospholipids become more viscous. This may result in increasing rigidity of the cell membrane and impedes membrane transport [26]. Moreover, the temperature influences growth and metabolic activity of bacteria [27]. Our study revealed strong correlation between specific ibuprofen removal rate and bacterial growth with the maximum at 30 °C (Figure 1b). Total inhibition of ibuprofen degradation at 42 °C is probably caused by denaturation of cell proteins. This reflects low optical density of bacteria at this temperature (Figure 1b). Moreover, the strong correlation between maximal degradation rate at 30 °C and maximal activity of hydroquinone monooxygenase and hydroxyquinol 1,2-dioxygenase at this temperature was observed (Figure 3a,c). Activity of phenol monooxygenase and catechol 1,2-dioxygenase, enzymes involved in degradation of another aromatic compounds by *Bacillus thuringiensis* B1(2015b), was also maximal at 30 °C (Figure 3b,d).

#### 2.1.2. Heavy Metals and Ibuprofen Degradation

In landfill sites, wastewaters, as well as in various natural environments, different types of contaminants are present. Contamination of the environment with aromatic compounds, including non-steroidal anti-inflammatory drugs, is often accompanied by the presence of heavy metals which may influence biodegradation processes. The effect of heavy metals on these processes depends on the kind of metal and its chemical and physical form such as separated-phase solids, soil-adsorbed species, colloidal solutions, soluble complexed species, or ions solutes [13,14]. The chemical and physical state of metals depends on the environmental conditions, such as pH, redox potential of the water phase, exchange capacity of soil, and organic matter content [11]. The influence of metals on biodegradation processes also depends on their impact on microorganisms [13]. Therefore, non-observed effect concentration (NOEC) of Cu(II), Cd (II), Co(II), Cr(VI), or Hg(II) for *Bacillus thuringiensis* B1(2015b), was determined [19]. The values obtained for the metals tested are listed in Table 1.

**Figure 4:**
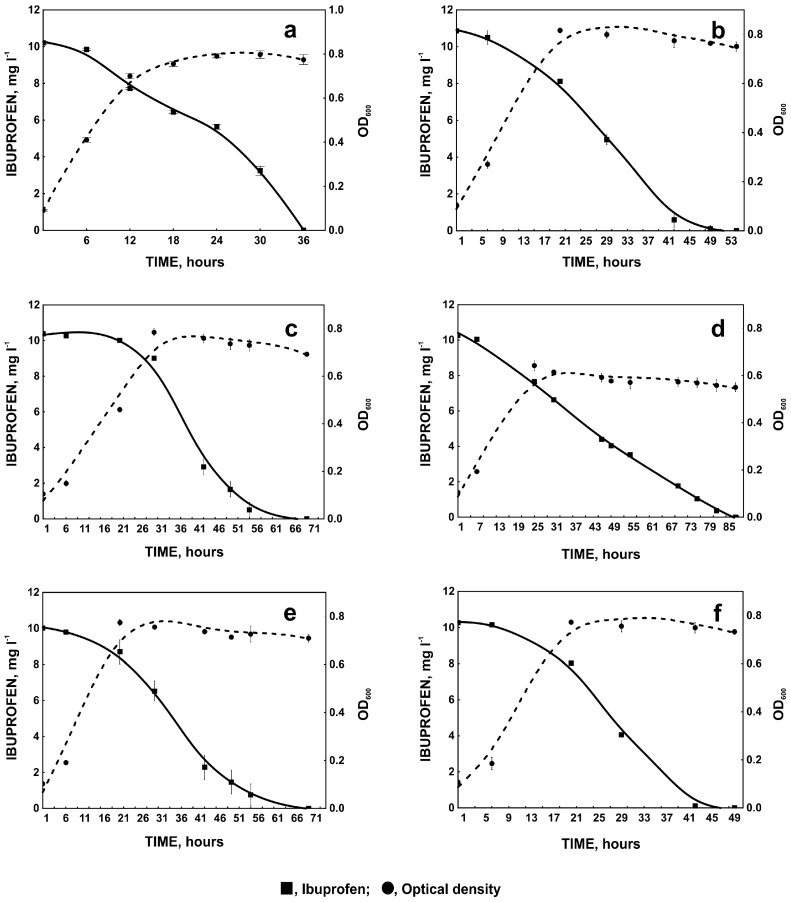
Biodegradation of ibuprofen by *Bacillus thuringiensis* B1(2015b) and changes in optical density of the control culture (**a**) and in the presence of metal ions: Cu(II) (**b**), Cd(II) (**c**), Co(II) (**d**), Cr(VI) (**e**), or Hg(II) (**f**). The data points represent the average of three independent experiments.

The highest inhibition of bacterial growth was observed in the presence of Hg^2+^ (NOEC = 0.3 µM). Mercury is a well-known pro-oxidant which exerts the oxidative stress via generation of hydrogen peroxide [28]. However, because B1(2015b) strain possess catalase activity, the toxicity of mercury could result from its ability to bind to the sulfhydryl groups of enzymes essential for microbial metabolism [11,19]. Although mercury ions inhibit growth of B1(2015b) strain, the NOEC of Hg(II) for this strain is significantly higher than the average concentration of mercury determined in sewage and other contaminated environments [29,30]. Addition of Hg(II) to the medium resulted in slower removal of ibuprofen in comparison to the control (without metal ion) (Figure 4a,f). This may suggest interaction of Hg(II) with enzymes engaged in ibuprofen degradation. Similar effect was observed by Said and Lewis [31] during degradation of 2,4-dichlorophenoxyacetic acid methyl ester by microbial consortium. In turn, Kuo and Genthner [13] observed stimulation of 2-chlorophenol and 3-chlorophenol biodegradation in the presence of metal ions at low concentration.

Low NOEC values for Cd(II) and Cu(II) (Table 1) indicate high sensitivity of *Bacillus thuringiensis* B1(2015b) to these metals. Cadmium is known to cause the oxidative stress and inhibit DNA repair processes [32]. The toxicity of copper may be the result of its high affinity for amines and carboxyl groups localized on cell surface of bacteria [33]. Copper ions may also interact with DNA and lead to the disorder of its helical structure. Like Cd(II), Cu(II) ions can bind to the thiol groups of various enzymes [33,34]. Despite the similar toxicity of Cd(II) and Cu(II) for B1(2015b) strain, cadmium ions significantly affect the degradation rate of ibuprofen (Figure 4a–c). This may result from the differences in the transport of these two ions into bacterial cell. Although copper is an essential trace element, it is toxic to cells at concentrations higher than physiological levels. Therefore, the intracellular concentration of copper is tightly controlled by the copper transport systems consist of P-type ATPases as CopA, CopB, copper chaperones, and chelators [35]. Due to the highly specific transporters, copper ions do not compete against other ions [36]. In turn, cadmium ions may enter bacterial cell by magnesium or manganese uptake systems, disturbing metal ion homeostasis in bacterial cells [37,38]. On the other hand, biodegradation processes are catalyzed by metalloenzymes that depend on divalent ions, such as Mn(II) or Fe(II) [39], and the disturbance of metal ions homeostasis may decrease the biodegradation efficiency. Inhibitory effects of cadmium may be also connected with its larger ionic radius, resulting in easier destabilization of degradative enzymes [38]. Inhibition of ibuprofen removal during first 31 h of incubation (Figure 4a,c) seems to confirm these speculations.

Co(II) and Cr(VI) ions were less toxic to B1(2015b) than the others (Table 1). Although Co(II) ions show lower toxicity and do not affect ibuprofen degradation during first hours of the experiment, their presence in the medium significantly extended the time necessary for the complete removal of the drug (Figure 4d). The inhibitory effect of cobalt was also observed by Yeom and Yoo [15] during degradation of benzene and toluene by *Alcaligenes xylosoxidans* Y234. Since, in the degradation of aromatic compounds, mono- and dioxygenases, the iron-binding proteins are engaged [39], cobalt may affect their activity by competition with iron during assembling the enzyme active site [40,41]. In the presence of 0.00082–2.56 µM Cr(VI), stimulation of bacterial growth was observed (Table 1). Although hexavalent chromium is very toxic to organisms, in the presence of glucose (when it is reduced to Cr(III) form) it stimulates glucose metabolism and in consequence bacterial growth [42,43]. At higher concentration Cr(III) is accumulated in the cell membrane and toxic for bacteria [42,44,45]. In the presence of hexavalent chromium degradation of ibuprofen was similar to this drug degradation in the presence of Cd(II). Cr(VI) does not influence the transport system of divalent cations, but it may oxidize divalent cations crucial for the activity of degradative enzymes [44,45].

#### 2.1.3. Degradation of Ibuprofen in the Presence of Selected Aromatic Compounds

**Figure 5:**
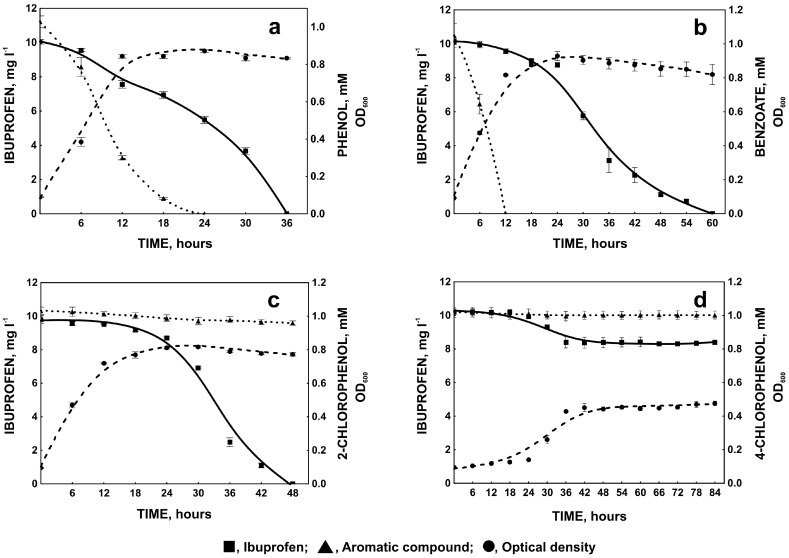
Biodegradation of ibuprofen by *Bacillus thuringiensis* B1(2015b), changes in optical density and degradation of phenol (**a**), benzoate (**b**); 2-chlorophenol (**c**); or 4-chlorophenol (**d**). The data points represent the average of three independent experiments.

Pharmaceutical pollutants coexist in the environment with other contaminants, such as aromatic compounds which enter the environment as a result of natural processes or human activity [17,46]. Due to the similar chemical structure to ibuprofen, these compounds may induce enzymes engaged in ibuprofen degradation. On the other hand, aromatic compounds may compete with ibuprofen for the position in the enzyme activity site, decreasing the degradation rate [17]. Therefore, the influence of phenol, benzoate, 2-chlorophenol, and 4-chlorophenol on ibuprofen degradation was examined. Of all the aromatic compounds used, only phenol does not influence ibuprofen degradation. Simultaneously with the degradation of ibuprofen, decrease in phenol concentration was observed (Figure 4a and Figure 5a). It was frequently demonstrated that phenol may be used as a carbon source during biodegradation of non-growth substances [47,48]. Nowak and Mrozik [49] observed degradation of 4-chlorphenol in the presence of phenol as a growth substrate. 4-chlorophenol like ibuprofen is a *para*-derivative of phenol. This may indicate that phenol did not inhibit degradation of its *para*-substituted derivatives [49]. On the other hand a decrease in the degradation of the phenol *para*-substituent in the presence of phenol was observed by Wang et al. [50]. This may result for different reasons. A decrease of the degradation rate may be caused by competition between substrates for the active site of the same enzyme. An increase in degradation may result from the accelerated induction of common degradative enzymes. If degradative enzymes belong to two different degradative pathways then the increase in degradation rate is caused with better biomass growth in the presence of growth substrate and in consequences with synthesis of essential reducing equivalent [49,50,51]. An example of the last situation is degradation of 4-chlorophenol in the presence of phenol observed by Nowak and Mrozik [49]. They showed that both 4-chlorophenol and phenol were cleaved by catechol 2,3-dioxygenase [49]. In our study we observed two different degradative pathway of phenol and ibuprofen. The key enzyme involved in phenol degradation by *Bacillus thuringiensis* B1(2015b) is catechol 1,2-dioxygenase whereas one of the enzyme in ibuprofen degradation pathway is hydroxyquinol 1,2-dioxygenase (Table 2) [20]. It is surprising that although phenol was degraded we did not observe stimulation of bacterial growth under this condition. In the presence of benzoate (Figure 4a and Figure 5b), ibuprofen degradation was slower. This may result from the competition between benzoate and ibuprofen for the active sites of degradative enzymes, connected with the similar acidic structure of these compounds. Our assumption is supported by the fact, that ibuprofen was not degraded until complete utilization of benzoate. Both, 2-chloro- and 4-chlorophenol were not decomposed by *Bacillus thuringiensis* B1(2015b) (Figure 5c,d). However, in the presence of 2-chlorophenol, slow degradation of ibuprofen was observed, while in the presence of 4-chlorophenol ibuprofen degradation was totally inhibited (Figure 5c,d). It is known that 2-chlorophenol is less toxic than other chlorophenols [52,53]. Very poor bacterial growth in the presence of 4-chlorophenol indicates high toxicity of this compound for *Bacillus thuringiensis* B1(2015b) (Figure 5d). Moreover, because of its chemical structure, ibuprofen may induce enzymes with high affinity to *para*-substituted derivatives. Hence, 4-chlorophenol could be bound more strongly by these enzymes and block their active sites.

### 2.2. Ibuprofen Degradation Pathway

**Figure 6:**
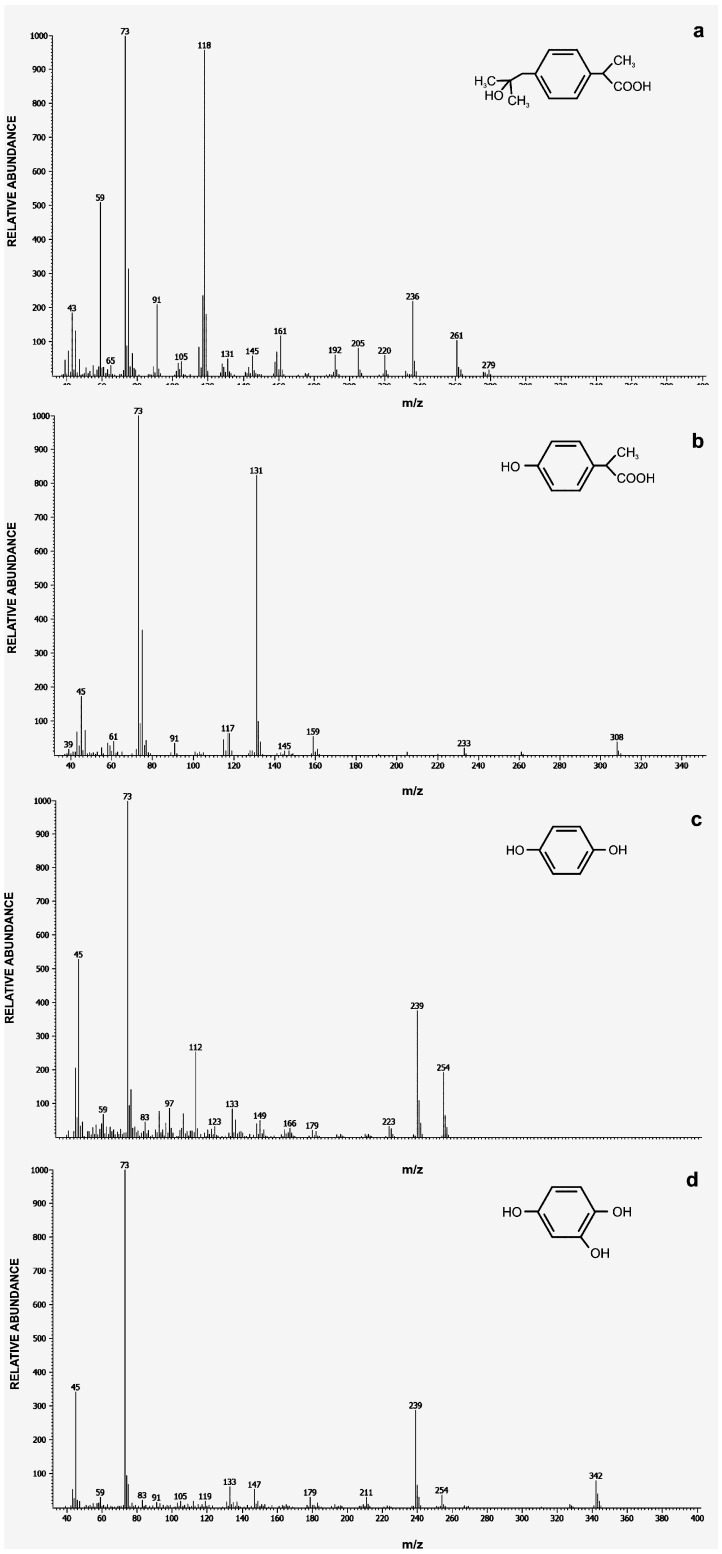
Spectra of putative metabolites identified via GC/MS analyses: 2-hydroxyibuprofen (**a**); 2-(4-hydroxyphenyl-) propionic acid (**b**); 1,4-hydroquinone (**c**); and 2-hydroxy-1,4-quinol (**d**).

**Figure 7:**
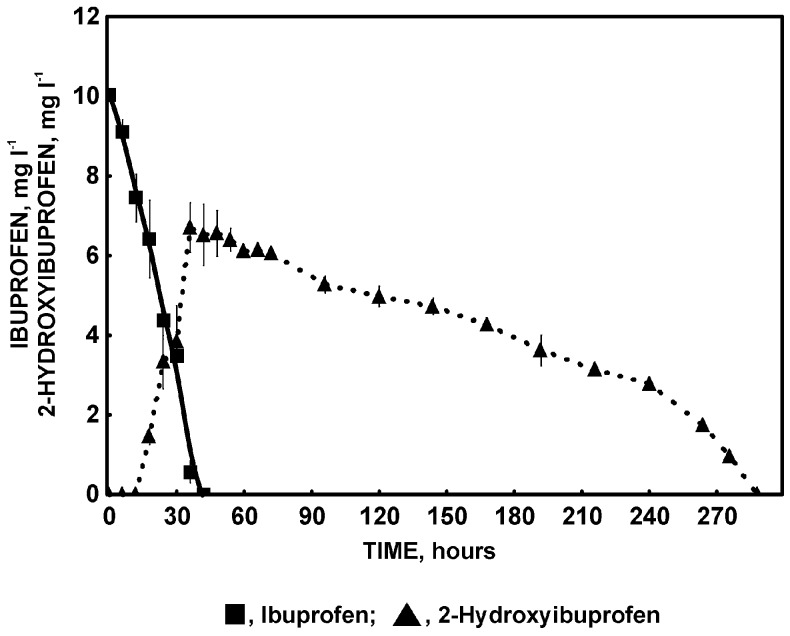
Appearance of 2-hydroxyibuprofen during ibuprofen degradation by *Bacillus thuringiensis* B1(2015b).

**Figure 8:**
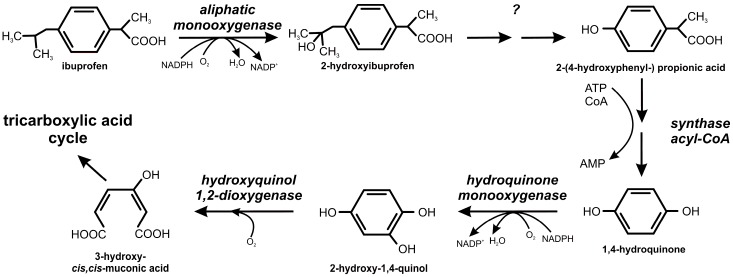
Ibuprofen degradation pathway in *Bacillus thuringiensis* B1(2015b).

So far the only pathway of ibuprofen biodegradation was described by Murdoch and Hay [5]. These authors suggested that catechols are the key metabolites in ibuprofen degradation and they are cleaved by extradiol dioxygenase to 5-formyl-2-hydroxy-7-methylocta-2,4-dienoic acid. Our studies showed that one of the most important step during ibuprofen degradation by *Bacillus thuringiensis* B1(2015b) is hydroxylation of both: aromatic ring and aliphatic chain of ibuprofen. This is confirmed by the high activity of aliphatic monooxygenases and phenol and hydroquinone monooxygenases (Table 2). GC-MS analysis of intermediates showed that the mass spectrum of the main intermediate had a molecular ion at *m*/*z* 59, 73, 118, 236 and 279. The obtained mass spectral fragmentation pattern after derivatization showed that the peak detected via GC-TOFMS was 2-hydroxyibuprofen (Figure 6a). This intermediate was probably formed as a result of aliphatic monooxygenase activity, appearing at 6 h of *Bacillus thuringiensis* B1(2015b) incubation and disappearing at 288 h of experimentation (Figure 7). Maximum concentration of 2-hydroxyibuprofen was observed at 42 h when ibuprofen was not detected. This may suggest that this step limits ibuprofen degradation rate. Hydroxylation is a typical, first reaction during ibuprofen degradation in both *Pro*- and *Eukaryotes* under oxic conditions [5,9,10,54]. The degradation product at *m*/*z* 45, 73, 131, 159, and 308 corresponded to 2-(4-hydroxyphenyl-) propionic acid (Figure 6b), which may be substrate for acyl-CoA synthase/thiolase (Table 2). The activity of this enzyme was also observed during ibuprofen degradation by *Sphingomonas* Ibu-2 [6]. Almeida et al. [8] also indicated involvement of this enzyme in ibuprofen decomposition by *Patulibacter* sp. strain I11. In the present studies mass spectra for other two intermediates from the extract of B1(2015b) culture were also detected. One spectrum corresponded to 1,4-hydroquinone (Figure 6c) and the second one to 2-hydroxyquinol (Figure 6d). 1,4-hydroquinone is a product of acyl-CoA synthase/thiolase activity and may be transform to 2-hydroxy-1,4-quinol by hydroquinone monooxygenase. The activity of these two enzymes was observed in the extract of culture (Table 2). Moreover, activity of hydroxyquinol 1,2-dioxygenase after the induction by ibuprofen was observed (Table 2) as in the presence of glucose alone the enzyme was not active at all. Hydroxyquinol 1,2-dioxygenase favorable binds 2-hydroxy-1,4-quinol and is responsible for *ortho* cleavage of this compound to 3-hydroxy-*cis*,*cis*-muconic acid [39]. It indicates that cleavage of 2-hydroxy-1,4-quinol by *Bacillus thuringiensis* B1(2015b) undergoes through the intradiol pathway. That is why we may speculate that degradation product of ibuprofen is 3-hydroxy-*cis,cis*-muconic acid which may be introduce to the central metabolism. The proposed ibuprofen degradation pathway in *Bacillus thuringiensis* B1(2015b) is presented in Figure 8. Hitherto it was only described as the extradiol cleavage of the ibuprofen derivative [5,7]. Therefore, our results provide the evidence for the functioning of the novel ibuprofen degradation pathway.

## 3. Materials and Methods

### 3.1. Bacterial Strain and Growth Conditions

*Bacillus thuringiensis* B1(2015b) was routinely cultivated in the nutrient broth at 30 °C for 24 h under oxic conditions with agitation at 130 rpm. After this, cells were harvested by centrifugation (5000× *g* at 4 °C for 15 min), washed with a fresh sterile medium and used as inoculum.

### 3.2. Ibuprofen Degradation Experiments

Degradation experiments were performed under aerobic conditions in 250 mL Erlenmeyer flasks containing 130 mL of the mineral salts medium [17] with glucose (1 g/L) inoculated with bacterial cells to a final optical density of about 0.1 at λ = 600 nm (OD_600_). Ibuprofen was added to a final concentration of 10 mg/L. Estimation of optimal temperature and pH for ibuprofen degradation was conducted in range 4–42 °C and 4.0–8.0, respectively. Bacterial growth was expressed as an optical density at 600 nm. To determine the effect of heavy metals and aromatic compounds on ibuprofen degradation, bacterial cultures were additionally supplemented with proper compound. Phenol, benzoic acid, 2-chlorophenol, and 4-chlorophenol were introduced into the cultures at a final concentration of 1 mM. Metal ions were added as CoSO_4_, Cd(NO_3_)_2_, K2Cr_2_O_7_, CuSO_4_ and HgCl_2_ at concentration equal to the estimated NOECs. All cultures were incubated with shaking (130 rpm) at 30 °C. The HPLC (High Performance Liquid Chromatography) analyses of each culture supernatant and measurements of the cultures growth were carried out 6 h.

For studying the microbial growth and ibuprofen degradation, the damped least-squares (DLS) method known as the Levenberg–Marquardt algorithm was used. The application of the Levenberg–Marquardt algorithm is in the least-squares curve fitting problem: given a set of *m* empirical datum pairs (*x_i_*, *y_i_*) of independent and dependent variables, find the parameter *θ* of the model curve *f(x*, *θ*) so that the sum of the squares of the deviations is minimized:

*g*(*θ*) = **Σ**_i=*m*_1__(*Y_i_ − f(θ*, *X_i_*))^2^ (1)

### 3.3. Determination of No-Observed-Effect Concentrations (NOECs) of Heavy Metals

In order to assess NOECs of Cu(II), Cd(II), Co(II), Cr(VI), and Hg(II) for *Bacillus thuringiensis* B1(2015b), Dunnett’s tests were performed. Initial concentration of each heavy metal tested was 50 mM, excluding Hg(II) (30 mM). The assays were performed using 96-well microtiter plates in three independent trials. Into each well, 200 µL of mineral salts medium with 1.25 g/L glucose, 50 µL of bacterial culture (optical density = 0.15), and an appropriate volume of metal ion solution was introduced. Cu(II), Cd(II), and Cr(VI) solutions were introduced to obtain the concentration range 0–8 mM; Hg(II) in the concentration range 0–4.8 mM while Co(II) was introduced to obtain a final concentration of 1.6 mM. After 24 h incubation in dark, at 30 °C, plates were subjected to the optical density analysis. The NOEC was defined as the highest toxic concentration that does not influence bacterial growth.

### 3.4. HPLC Analysis

Cultures samples were collected and analyzed every 6 h during first three days of the experiment (72 h) and then every 24 h tthereafter. The concentration of ibuprofen, 2-hydroxyibuprofen, phenol, benzoic acid, 2-chlorophenol, and 4-chlorophenol was determined with the HPLC technique using a Merck Hitachi (Darmstadt, Germany) HPLC reversed-phase chromatograph equipped with Ascentis Express^®^ C18 HPLC Column (100 × 4.6 mm), pre-column Opti-Solw^®^ EXP, and UV-VIS DAD detector. The mobile phase consisted of acetonitrile and 1% acetic acid (50:50 *v*/*v*) at a flow rate of 1 mL/min. For phenol, benzoic acid, 2-chlorophenol, and 4-chlorophenol, the detection wavelength was set at 260 nm, and for ibuprofen and 2-hydroxyibuprofen at 240 nm [19]. Ibuprofen, 2-hydroxyibuprofen, phenol, benzoic acid, 2-chlorophenol, and 4-chlorophenol concentrations were identified by comparing the HPLC retention times and the UV-VIS spectra with those of the external standards.

### 3.5. Enzyme Assays

After five days of incubation, *Bacillus thuringiensis* B1(2015b) cells were transferred to fresh mineral salts medium supplemented with 10 mg/L ibuprofen and 1 g/L glucose. After 24 h, cells were harvested by centrifugation (4500× *g* for 15 min at 4 °C) and the pellet was washed with 50 mM phosphate buffer, pH 7.0, and resuspended in the same buffer. Cell-free extracts were prepared by sonication of the whole cells suspension (6 times for 15 s) and centrifuged at 9000× *g* for 30 min at 4 °C. Clear supernatant was used as a crude cell extract for enzyme assays. The enzymes for enzyme activity analysis were selected based on the literature data indicating enzymes involved in the decomposition of the aromatic structure under aerobic conditions.

Activity of aromatic monooxygenases was determined spectrophotometrically by measuring NADH oxidation rate (ε_340_ = 6220/M cm) [55].

Aliphatic monooxygenase activity was monitored by measuring NADH oxidation (ε_340_ = 220/M cm) in sealed assay vials containing 5 mL of methane, pentane, butane/isobutene, or propane/butane (70:30 *v*/*v*), phosphoric buffer pH 7.4 (50 mM), NADH (200 µM), FAD (8.8 µM), and crude enzyme extracts in a total volume of 2 mL [56].

The activity of catechol 1,2-dioxygenase was measured spectrophotometrically by the formation of cis,cis-muconic acid at 260 nm (ε_260_ = 16,800/M cm). In order to determine catechol 2,3-dioxygenase activity, the formation of 2-hydroxymuconic semialdehyde was measured at 375 nm (ε_375_ = 36,000/M cm) [51]. The activity of protocatechuate 3,4-dioxygenase was assayed by measuring the oxygen consumption [57]. The activity of protocatechuate 4,5-dioxygenase was measured spectrophotometrically by the formation of 2-hydroxy-4-carboxymuconic semialdehyde at 410 nm (ε_410_ = 9700/M cm) [51]. In order to determine gentisate 1,2-dioxygenase activity, the formation of maleylpyruvate was measured at 330 nm (ε_330_ = 10,800/M cm) [58]. The activity of hydroxyquinol 1,2-dioxygenase was measured spectrophotometrically by the formation of maleylacetate at 243 nm (ε_243_ = 44,520/M cm) [59]. The activity of hydroquinone 1,2-dioxygenase was measured spectrophotometrically by the formation of 4-hydroxymuconic semialdehyde at 320 nm (ε_320_ = 11,000/M cm) [60].

Acetyl-coenzyme A synthethase activity was measured spectrophotometrically based on NAD^+^ reduction to NADH at 340 nm (NADH: ε_340_ = 6220/M cm) with combination of malate dehydrogenase and citrate synthase [61,62]. The enzymatic assay mixture contains: 50 µL L-malate (50 mM), 50 µL ATP (20 mM), 50 µL MgCl_2_ (50mM), 50 µL coenzyme A (2 mM), 50 µL NAD^+^ (50 mM), 50 µL malate dehydrogenase, 50 µL citrate synthetase, 450 µL phosphoric buffer (100 mM), 100 µL sodium acetate (1 M), and 100 µL crude extract in total mixture volume of 1 mL.

The activity of laccases and peroxidases was measured spectrophotometrically based on the syringaldazine oxidation product formation at 525 nm (ε_525_ = 65,000/M cm). The enzymatic mixture contains: 110 µL syringaldazine (0.05 mM in 60% ethanol), 100 µL crude extract, 1 µL catalase (10 mg/mL) in case of laccase, or 100 μL H_2_O_2_ (200 mM) for peroxidases and mixtures were filled up with phosphoric buffer pH 7.4 (50 mM) to total mixture volume of 1 mL [63].

One unit of the enzyme activity was defined as the amount of enzyme required to generate 1 µmol of product per minute. Protein concentration in the crude extract was determined by the Bradford method using bovine serum albumin as a standard [51].

### 3.6. Intermediates Identification

Cultures samples, in the volume of 100 mL, were collected every seven days for six weeks. All samples were centrifuged and acidified with hydrochloric acid to pH 3.0. Next, samples were combined and extracted. The obtained extract was divided into two 300 mL fractions. One of them was frozen in the liquid nitrogen, lyophilized and extracted with 10 mL of hexane (HPLC grade) (2×) and 10 mL of toluene (HPLC grade) (2×), mixed and evaporated. Second fraction was extracted twice, at first with hexane (HPLC grade) and then with toluene (HPLC grade) in the volume ratio 3:1 (*v*/*v*) in both cases. Obtained extracts were mixed and evaporated. All cultures and extracts were prepared in triplicate.

To each sample, 0.1 mL of derivatization reagent MTBSTFA with 1% t-BDMCS was added. After incubation at 60 °C for 30 min, the sample was dissolved in 1 mL of hexane. To qualitative analysis the PEGASUS 4D GCxGC-TOFMS gas chromatograph (LECO Corp., St. Joseph, MI, USA) connected to a BPX5 (5% phenyl equivalent, 28 m × 0.25 mm; 0.25 μm) capillary column (SGE Int., Melbourne, Australia) was used. The ion source and transfer line temperature were set at 250 °C. Helium was used as carrier gas, with a flow of 1.0 mL/min. After splitless injection of 1 μL of the sample, the oven temperature was set and maintained for 2 min at 40 °C, then it has been applied a heating ramp to 300 °C at a rate of 12 °C/min and maintaining the temperature of the oven for 15 min. The ionization source operated in the positive ion mode (electron energy: 70 V) and the acquisition rate was set at 10 spectra/s. The ibuprofen metabolites were identified by its mass spectra, as well as by comparison of the retention time of the analytical standards.

## Figures and Tables

**Table 1:** Effect of metal ions on *Bacillus thuringiensis* B1(2015b) growth.

Metal Ion	Metal Ion Concentration (mM)	Optical Density (OD_600_)	NOEC (mM)
Cu^2+^	0.00000082	0.150 ± 0.040	0.00256
	0.0000041	0.146 ± 0.026	
	0.00002048	0.138 ± 0.033	
	0.0001024	0.139 ± 0.019	
	0.000512	0.145 ± 0.016	
	0.00256	0.156 ± 0.012	
	0.128 *	0.092 ± 0.015	
	0.064 *	0.109 ± 0.015	
	0.32 *	0.094 ± 0.034	
	1.6 *	0.077 ± 0.019	
Cd^2+^	0.000001	0.111 ± 0.009	0.003
	0.000004	0.106 ± 0.028	
	0.00002	0.113 ± 0.025	
	0.000102	0.118 ± 0.032	
	0.001	0.120 ± 0.034	
	0.003	0.117 ± 0.030	
	0.013 *	0.108 ± 0.001	
	0.064 *	0.119 ± 0.011	
	0.32 *	0.124 ± 0.027	
	1.6 *	0.079 ± 0.008	
	8.0 *	0.061 ± 0.008	
Co^2+^	0.17	0.149 ± 0.008	0.21
	0.21	0.162 ± 0.015	
	0.27 *	0.120 ± 0.014	
	0.34 *	0.108 ± 0.022	
	0.42 *	0.095 ± 0.010	
	0.52 *	0.050 ± 0.016	
	0.66 *	0.035 ± 0.009	
	0.82 *	0.019 ± 0.008	
	1.02 *	0.020 ± 0.004	
	1.28 *	0.017 ± 0.008	
	1.60 *	0.017 ± 0.008	
Cr^6+^	0.00000082 *	0.079 ± 0.021	0.32
	0.0000041 *	0.092 ± 0.030	
	0.00002048 *	0.095 ± 0.030	
	0.0001024 *	0.127 ± 0.023	
	0.000512 *	0.170 ± 0.021	
	0.00256 *	0.138 ± 0.021	
	0.128	0.065 ± 0.008	
	0.064	0.009 ± 0.004	
	0.32	0.004 ± 0.003	
	1.6 *	0.003 ± 0.003	
	8.0 *	0.000 ± 0.000	
Hg^2+^	0.000000492	0.104 ± 0.015	0.000307
	0.000002	0.103 ± 0.023	
	0.000012	0.113 ± 0.002	
	0.000061	0.108 ± 0.006	
	0.000307	0.121 ± 0.013	
	0.001536 *	0.047 ± 0.014	
	0.00768 *	0.000 ± 0.000	
	0.0384 *	0.000 ± 0.000	
	0.192 *	0.000 ± 0.000	
	0.96 *	0.000 ± 0.000	
	4.8 *	0.000 ± 0.000	

* Statistically different from control group (one-tailed Dunnett’s test, *p* < 0.05).

**Table 2:** Specific activity of enzymes after ibuprofen induction.

Enzyme	Specific Enzyme Activity (mU/mg Protein)	Enzyme	Specific Enzyme Activity (mU/mg Protein)
Methane monooxygenase	831.39 ± 167.97	Catechol 2,3-dioxygenase	0.00 ± 0.00
Butane/isobutane monooxygenase	150.94 ± 21.95	Protocatechuate 3,4-dioxygenase	0.00 ± 0.00
Propane/butane monooxygenase	311.77 ± 62.99	Protocatechuate 4,5-dioxygenase	0.00 ± 0.00
Pentane monooxygenase	0.00 ± 0.00	Gentisate 1,2-dioxygenase	0.00 ± 0.00
Phenol monooxygenase	77.40 ± 4.50	Hydroxyquinol 1,2-dioxygenase	99.12 ± 12.92
Hydroquinone monooxygenase	102.68 ± 14.24	Hydroquinone 1,2-dioxygenase	0.00 ± 0.00
Catechol 1,2-dioxygenase	34.99 ± 2.91	Acetyl-coenzyme A synthetase	249.47 ± 24.16
Laccase	0.00 ± 0.00	Peroxidase	0.00 ± 0.00
